# The Applications of Artificial Intelligence for Assessing Fall Risk: Systematic Review

**DOI:** 10.2196/54934

**Published:** 2024-04-29

**Authors:** Ana González-Castro, Raquel Leirós-Rodríguez, Camino Prada-García, José Alberto Benítez-Andrades

**Affiliations:** 1 Nursing and Physical Therapy Department Universidad de León Ponferrada Spain; 2 SALBIS Research Group Nursing and Physical Therapy Department Universidad de León Ponferrada Spain; 3 Department of Preventive Medicine and Public Health Universidad de Valladolid Valladolid Spain; 4 SALBIS Research Group Department of Electric, Systems and Automatics Engineering Universidad de León León Spain

**Keywords:** machine learning, accidental falls, public health, patient care, artificial intelligence, AI, fall risk

## Abstract

**Background:**

Falls and their consequences are a serious public health problem worldwide. Each year, 37.3 million falls requiring medical attention occur. Therefore, the analysis of fall risk is of great importance for prevention. Artificial intelligence (AI) represents an innovative tool for creating predictive statistical models of fall risk through data analysis.

**Objective:**

The aim of this review was to analyze the available evidence on the applications of AI in the analysis of data related to postural control and fall risk.

**Methods:**

A literature search was conducted in 6 databases with the following inclusion criteria: the articles had to be published within the last 5 years (from 2018 to 2024), they had to apply some method of AI, AI analyses had to be applied to data from samples consisting of humans, and the analyzed sample had to consist of individuals with independent walking with or without the assistance of external orthopedic devices.

**Results:**

We obtained a total of 3858 articles, of which 22 were finally selected. Data extraction for subsequent analysis varied in the different studies: 82% (18/22) of them extracted data through tests or functional assessments, and the remaining 18% (4/22) of them extracted through existing medical records. Different AI techniques were used throughout the articles. All the research included in the review obtained accuracy values of >70% in the predictive models obtained through AI.

**Conclusions:**

The use of AI proves to be a valuable tool for creating predictive models of fall risk. The use of this tool could have a significant socioeconomic impact as it enables the development of low-cost predictive models with a high level of accuracy.

**Trial Registration:**

PROSPERO CRD42023443277; https://tinyurl.com/4sb72ssv

## Introduction

### Background

According to alarming figures reported by the World Health Organization in 2021, falls cause 37.3 million injuries annually that require medical attention and result in 684,000 deaths [1]. These figures indicate a significant impact of falls on the health care system and on society, both directly and indirectly [2,3].

Life expectancy has progressively increased over the years, leading to an aging population [4]. By 2050, it is estimated that 16% of the population will be >65 years of age. In this group, the incidence of falls has steadily risen, becoming the leading cause of accidental injury and death (accounting for 55.8% of such deaths, according to some research) [5,6]. It is estimated that 30% of this population falls at least once a year, negatively impacting their physical and psychological well-being [7,8].

Physically, falls are often associated with severe complications that can lead to extended hospitalizations [9]. These hospitalizations are usually due to serious injuries, often cranioencephalic trauma, fractures, or soft tissue injuries [10,11]. Psychologically, falls among the older adult population tend to result in self-imposed limitations due to the fear of falling again [10,12]. These limitations lead to social isolation as individuals avoid participating in activities or even individual mobility [13]. Consequently, falls can lead to psychological conditions such as anxiety and depression [14,15]. Numerous research studies on the risk of falls are currently underway, with ongoing investigations into various innovations and intervention ideas [16-19]. These studies encompass the identification of fall risk factors [20,21], strategies for prevention [22,23], and the outcomes following rehabilitation [23,24].

In the health care field, artificial intelligence (AI) is characterized by data management and processing, offering new possibilities to the health care paradigm [24]. Some applications of AI in the health care domain include assessing tumor interaction processes [25], serving as a tool for image-based diagnostics [26,27], participating in virus detection [28], and, most importantly, as a statistical and predictive method [29-32].

Several publications have combined AI techniques to address health care issues [33-35]. Within the field of predictive models, it is important to understand certain differentiations. In AI, we have machine learning and deep learning [36-38]. Machine learning encompasses a set of techniques applied to data and can be done in a supervised or unsupervised manner [39,40]. On the other hand, deep learning is typically used to work with larger data sets compared to machine learning, and its computational cost is higher [41,42].

Some examples of AI techniques include the gradient boosting machine [43], learning method, and the long short-term memory (LSTM) [44] and the convolutional neural network (CNN) [45], all of them are deep learning methods.

### Objectives

For all the reasons mentioned in the preceding section, it was considered necessary to conduct a systematic review to analyze the scientific evidence of AI applications in the analysis of data related to postural control and the risk of falls.

## Methods

### Data Sources and Searches

This systematic review and meta-analysis were prospectively registered on PROSPERO (ID CRD42023443277) and followed the Meta-Analyses of Observational Studies in Epidemiology checklist [46] and the recommendations of the Cochrane Collaboration [47].

The search was conducted in January 2024 on the following databases: PubMed, Scopus, ScienceDirect, Web of Science, CINAHL, and Cochrane Library. The Medical Subject Headings (MeSH) terms used for the search included *machine learning*, *artificial intelligent*, *accidental falls*, *rehabilitation*, and *physical therapy specialty*. The terms “predictive model” and “algorithms” were also used. These terms were combined using the Boolean operators AND and OR (Textbox 1).

**Search strategy according to the focused question.**

**PubMed**

- (“machine learning”[MeSH Terms] OR “artificial intelligent”[MeSH Terms]) AND “accidental falls”[MeSH Terms]
- (“machine learning”[MeSH Terms] OR “artificial intelligent”) AND (“rehabilitation”[MeSH Terms] OR “physical therapy specialty”[MeSH Terms])
- “accidental falls” [Title/Abstract] AND “algorithms” [Title/Abstract]
- “accidental falls”[Title/Abstract] AND “predictive model” [Title/Abstract]

**Scopus**

- TITLE-ABS-KEY (“machine learning” OR “artificial intelligent”) AND TITLE-ABS-KEY (“accidental falls”)
- TITLE-ABS-KEY (“machine learning” OR “artificial intelligent”) AND TITLE-ABS-KEY (“rehabilitation” OR “physical therapy specialty”)
- TITLE-ABS-KEY (“accidental falls” AND “algorithms”)
- TITLE-ABS-KEY (“accidental falls” AND “predictive model”)

**ScienceDirect**

- Title, abstract, keywords: (“machine learning” OR “artificial intelligent”) AND “accidental falls”
- Title, abstract, keywords: (“machine learning” OR “artificial intelligent”) AND (“rehabilitation” OR “physical therapy specialty”)
- Title, abstract, keywords: (“accidental falls” AND “algorithms”)
- Title, abstract, keywords: (“accidental falls” AND “predictive model”)

**Web of Science**

- TS=(“machine learning” OR “artificial intelligent”) AND TS=“accidental falls”
- TS=(“machine learning” OR “artificial intelligent”) AND TS= (“rehabilitation” OR “physical therapy specialty”)
- AB= (“accidental falls” AND “algorithms”)
- AB= (“accidental falls” AND “predictive model”)

**CINAHL**

- (MH “machine learning” OR MH “artificial intelligent”) AND MH “accidental falls”
- (MH “machine learning” OR MH “artificial intelligent”) AND (MH “rehabilitation” OR MH “physical therapy specialty”)
- (AB “accidental falls”) AND (AB “algorithms”)
- (AB “accidental falls”) AND (AB “predictive model”)

**Cochrane Library**

- (“machine learning” OR “artificial intelligent”) in Title Abstract Keyword AND “accidental falls” in Title Abstract Keyword
- (“machine learning” OR “artificial intelligent”) in Title Abstract Keyword AND (“rehabilitation” OR “physical therapy specialty”) in Title Abstract Keyword
- “accidental falls” in Title Abstract Keyword AND “algorithms” in Title Abstract Keyword
- “accidental falls” in Title Abstract Keyword AND “predictive model” in Title Abstract Keyword

### Study Selection

After removing duplicates, 2 reviewers (AGC and RLR) independently screened articles for eligibility. In the case of disagreement, a third reviewer (JABA) finally decided whether the study should be included or not. We calculated the κ coefficient and percentage agreement scores to assess reliability before any consensus and estimated the interrater reliability using κ. Interrater reliability was estimated using κ>0.7 indicating a high level of agreement between the reviewers, κ of 0.5 to 0.7 indicating a moderate level of agreement, and κ<0.5 indicating a low level of agreement [48].

For the selection of results, the inclusion criteria were established as follows: (1) articles should have been published in the last 5 years (from 2018 to the present); (2) they must apply some AI method; (3) AI analyses should be applied to data from samples of humans; and (4) the sample analyzed should consist of people with independent walking, with or without the use of external orthopedic devices.

After screening the data, extracting, obtaining, and screening the titles and abstracts for inclusion criteria, the selected abstracts were obtained in full texts. Titles and abstracts lacking sufficient information regarding inclusion criteria were also obtained as full texts. Full-text articles were selected in case of compliance with inclusion criteria by the 2 reviewers using a data extraction form.

### Data Extraction and Quality Assessment

The 2 reviewers mentioned independently extracting data from the included studies using a customized data extraction table in Excel (Microsoft Corporation). In case of disagreement, both reviewers debated until an agreement was reached.

The data extracted from the included articles for further analysis were: demographic information (title, authors, journal, and year), characteristics of the sample (age, inclusion and exclusion criteria, and number of participants), study-specific parameters (study type, AI techniques applied, and data analyzed), and the results obtained. Tables were used to describe both the studies’ characteristics and the extracted data.

### Assessment of Risk of Bias

The methodological quality of the selected articles was evaluated using the Critical Review Form for Quantitative Studies [49]. The ROBINS-E (Risk of Bias in Nonrandomized Studies of Exposures) tool was used to evaluate the risk of bias [50].

## Results

### Characteristics of the Selected Studies

**Figure 1:**
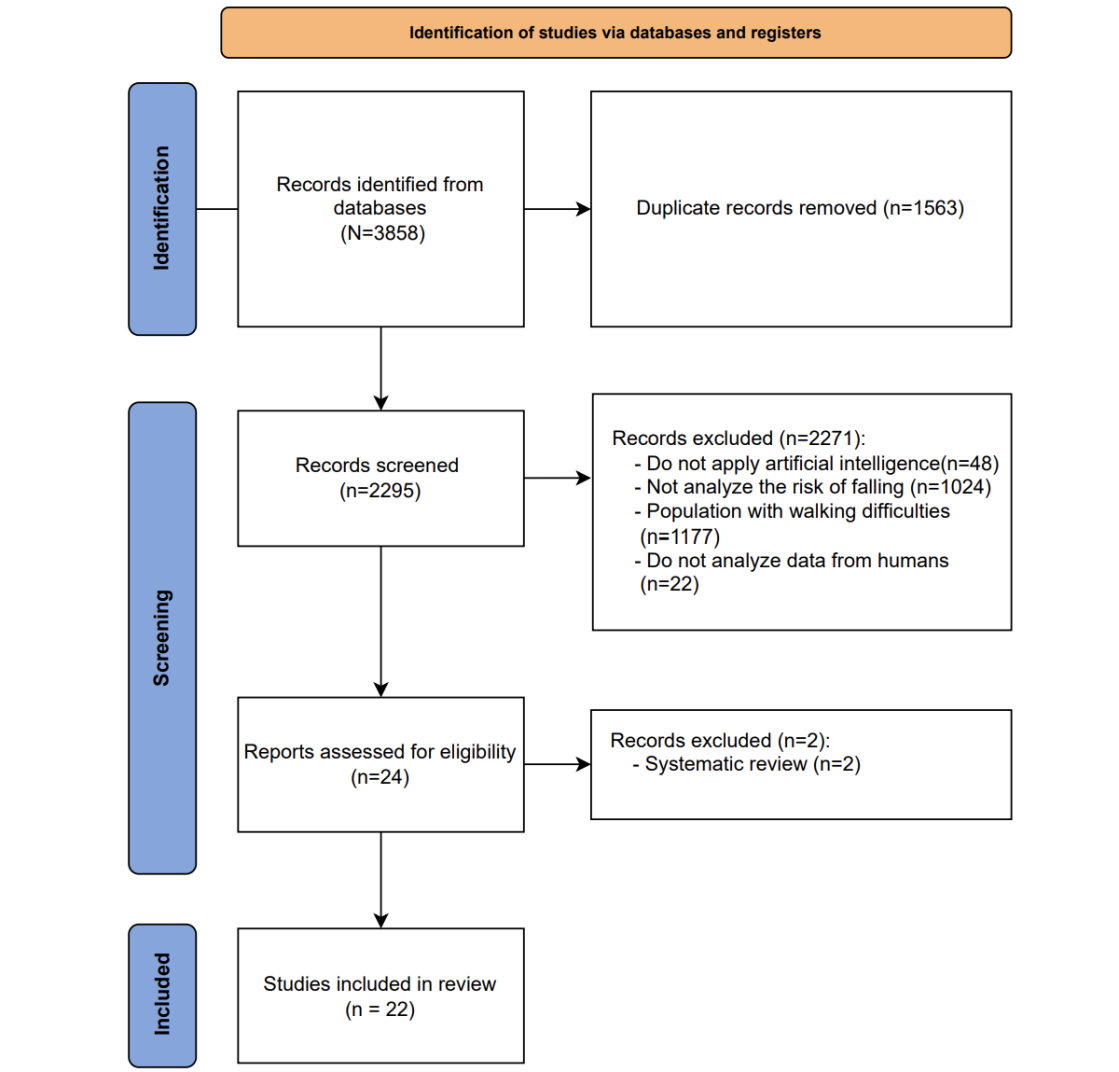
Identification of studies via databases and registers.

A total of 3858 articles were initially retrieved, with 1563 duplicates removed. From the remaining 2295 articles, 2271 were excluded based on the initial selection criteria, leaving 24 articles for the subsequent analysis. In this second analysis, 2 articles were removed as they were systematic reviews, and 22 articles were finally selected [51-72] (Figure 1). After the first reading of all candidate full texts, the kappa score for inclusion of the results of reviewers 1 and 2 was 0.98, indicating a very high level of agreement.

The methodological quality of the 22 analyzed studies (Table S1 in Multimedia Appendix 1 [51,52,54,56,58,59,61,63,64, 69,70,72]) ranged from 11 points in 2 (9.1%) studies [52,65] to 16 points in 7 (32%) studies [53,54,56,63,69-71].

### Study Characteristics and Risk of Bias

All the selected articles were cross-sectional observational studies (Table 1).

In total, 34 characteristics affecting the risk of falls were extracted and classified into high fall-risk and low fall-risk groups with the largest sample sizes significantly differing from the rest. Studies based on data collected from various health care systems had larger sample sizes, ranging from 22,515 to 265,225 participants [60,65,67]. In contrast, studies that applied some form of evaluation test had sample sizes ranging from 8 participants [56] to 746 participants [55].

It is worth noting the various studies conducted by Dubois et al [54,72], whose publications on fall risk and machine learning started in 2018 and progressed until 2021. A total of 9.1% (2/22) of the articles by this author were included in the final selection [54,72]. Both articles used samples with the same characteristics, even though the first one was composed of 43 participants [54] and the last one had 30 participants [72]. All 86.4% (19/22) of the articles used samples of individuals aged ≥65 years [51-60,62-65,68-72]. In the remaining 13.6% (3/22) of the articles, the ages ranged between 16 and 62 years [61,66,67].

Althobaiti et al [61] used a sample of participants between the ages of 19 and 35 years for their research, where these participants had to reproduce examples of falls for subsequent analysis. In 2022, Ladios-Martin et al [67] extracted medical data from participants aged >16 years for their research. Finally, in 2023, the study by Maray et al [66] used 3 types of samples, with ages ranging from 21 to 62 years. Among the 22 selected articles, only 1 (4.5%) of them did not describe the characteristics of its sample [52].

Finally, regarding the sex of the samples, 13.6% (3/22) of the articles specified in the characteristics of their samples that only female individuals were included among their participants [53,59,70].

**Table 1:** Methodological characteristics of results analyzed.

Study	Age range (mean; years)	Assessment procedures	AI^a^ techniques	Results
Nait Aicha et al [51], (2018)	65-99 (75.3)	Over the following 6 months, they had monthly consultations to monitor the incidence of falls.	Classification of participants into fall and nonfall categories and creation of an ML^b^ model that predicts falls from the accelerometer data.	A total of 34.1% of the participants experienced at least one fall. ML applied to the accelerometric data showed a predictive model of comparable accuracy to conventional fall risk assessment models.
Althobaiti et al [61], (2020)	19-34 (nd^c^)	Participants performed 6 ADLs^d^ and 9 falls on a foam landing mat. Accelerometer data were transmitted via Bluetooth and recorded continuously.	After extracting the signals from the accelerometers, the data were used to train ML models.	The ML models created can predict and detect falls with high accuracy. The supervised classification experiments achieved an *F*_1_-score of 98.4% for distinguishing between falls and nonfalls, and an *F*_1_-score of 88.1% for distinguishing between ADLs.
Dubois et al [54], (2018)	61-93 (83)	The TUG^e^ test was administered 3 times, with 3-5 minutes of rest between attempts. The best result was used to categorize participants into low and high risk of falling.	The images were analyzed with a processing algorithm, and ML techniques were applied to predict the risk of falls.	All participants were classified using a combination of walking speed, stride length, and sitting speed. The algorithm used was used to correctly classify them according to their risk of falling and to assess their progress.
Dubois et al [72], (2021)	61-93 (83.3)	A first assessment, including the Tinetti and TUG tests, was conducted by a physiotherapist to identify patients who are “high risk.” Daily activity was monitored for 8 hours.	Different parameters, such as walking speed, sitting speed, and total sitting time, were analyzed. The data were analyzed by combining statistics and ML algorithms.	The results obtained from the algorithms were compared with those obtained by physiotherapists using the Tinetti and TUG tests. Step length and sit-to-stand time could complement the assessment tools and improve the risk of falls.
Eichler et al [68], (2022)	65-nd (nd)	BBS^f^ (consisting of 14 tasks). A total of 2 physiotherapists independently scored each participant on the 14 tasks. The number of participants varied across tasks due to differences in performance abilities.	ML to predict the risk of falling by categorizing it as “high,” “medium,” or “low.” Initially, the movements of the participants were tracked using deep learning applied to the camera while they performed the BBS. Then, an algorithm predicted the BBS score, leading to the final risk classification.	The results obtained through deep learning were compared with the scores given by the physiotherapists, and the overall accuracy was 75.5% correct. The ML algorithm reduced the false-negative rate by adjusting the thresholds. The most predictive features were: 360° turn, alternating feet on the step, chair transfer, and reaching forward with the arm extended.
Ferrete et al [52], (2019)	nd	Participants were instructed to act out falls.	A total of 2 deep learning approaches were explored: the classifier based on ASM^g^ and CNN^h^.	The CNN demonstrated 92.7% accuracy in differentiating normal gait, prefall condition, and fall situation, considering the prefall step, and 100% when not considering the prefall step.
Gillain et al [64], (2019)	65-89 (71.3)	Participants underwent a physical and functional assessment, which included the evaluation of gait speed, stride length, frequency, symmetry, regularity, and minimum toe separation. These variables were recorded under conditions of comfortable walking, fast walking, and dual-task walking.	A supervised ML algorithm (J48) was applied to the recorded data.	A classification tree was obtained that correctly identifies 80% of future falls based on gait parameters, gender, leg length, and age. Accuracy was 84%, sensitivity was 80%, specificity was 87%, positive predictive value was 78%, and negative predictive value was 88%.
Greene et al [70], (2021)	nd (74.7)	The test lasted 30 seconds and was conducted with both eyes open and closed. A comprehensive geriatric assessment and the BBS were used to obtain risk factors for each participant. Data were collected using the Kinesis Balance FP^i^ application, and a questionnaire on the socio-demographic data of the participants was administered through the app.	The ML algorithm assessed balance and the risk of falling based on previously collected data.	The use of ML models for balance and fall risk assessment could improve the approach to falls, including prescribing personalized prevention programs and monitoring progress. This would improve the remote care system with more targeted and digitized initiatives.
Hauth et al [56], (2021)	69-82 (nd)	IMUs^j^ measured linear and angular accelerations in the mediolateral, anteroposterior, and longitudinal axes. Participants wore a wrist-mounted voice recorder to indicate when the event occurred and to provide context descriptions.	Customized algorithms were used to integrate the data from the IMUs in 6 dimensions to obtain the position, orientation, and velocity of the foot. Model performance was evaluated in terms of AUROC^k^ and AUPR^l^.	Algorithms for data integration could be used retrospectively to track the occurrence of falls or loss of balance events. The model showed discriminatory ability to differentiate a fall from a normal walking event. The best model achieved an AUROC ≥0.87 for nonparticipants.
Hsu et al [58], (2020)	nd	All participants were assessed for fall risk using the MFS^m^ upon admission.	The XGB^n^ predictive model was applied, and its performance was compared with that of the MFS.	The XGB model is more sensitive than the MFS, and therefore it is more appropriate for assessing the risk of falls.
Hu et al [59], (2020)	nd	Participants underwent treadmill walking and MCT^o^ with small, medium, and large perturbations. They were classified into a high-balance group and a low-balance group based on the MCT results.	ML analysis: 5-fold cross-validation where the algorithms were repeatedly randomly trained. Using the GBM^p^ algorithm, participants were classified into high and low equilibrium through analysis of the accelerometric data.	The feasibility of ML was demonstrated by matching accelerometric data and predicting balance dysfunctions. This method was comparable to the data obtained from instrumented balance tests and was also less expensive.
Kim et al [63], (2019)	nd	All participants simulated various types of falls, resulting in a total of 282 scenes constituting the database. Falls, including forward, backward, and sideways motions, were replicated. Additionally, images without falls depicted actions such as sitting on a chair or lying on a bed.	The application of ML: The RF^q^ system was used as a data management and predictive tool. A total of 200 decision trees were used, with 25 trees deep for each tree.	Improved detection of false fall data. The model combining accelerometric data and vision data gave promising results. The application of ML achieved good generalization of the obtained data. Accuracy of the proposed method was 90%.
Ladios-Martin et al [67], (2022)	16-106 (nd)	In total, 91 variables related to the risk of falls were selected. A total of 2 models were created: model A included the variable FP, while model B did not.	The performance of 10 algorithms was evaluated. Once the final models were created, they were compared with each other by evaluating their performances.	The performance of the model that included the FP variable performed better than that of the one that did not include it. Using AI techniques and the FP variable, a high-performance model is obtained that can be exported to other contexts.
Lo et al [65], (2019)	65-nd (nd)	Researchers established which patients were at risk of falls based on existing medical record data.	ML techniques (RF algorithm) were used to build a predictive model of fall risk.	The risk of falls is affected by a large number of factors (age, clinical diagnoses, daily habits, environment, and hygiene). ML makes it possible to predict the risk of falls and rank the importance of each risk factor.
Lockhart et al [57], (2021)	56-9 (74.3)	Participants conducted 10-meter walks. Out of 127 participants, 79.5%) did not experience falls, while 20.5%) did.	A total of 3 types of deep learning techniques were applied, and a predictive model was created with the data from the 127 participants (RF, RF with feature engineering, and RF with feature engineering and linear and nonlinear variables). The accuracy of the model was tested with the remaining 34.6%) participants with a 6-month follow-up of their fall history.	The IMU was a good tool for the identification of falls risk. Data analysis through AI obtained very good predictive results.
Maray et al [66], (2023)	21-65 (nd)	All participants performed fall representations.	Deep learning was used to match the data extracted from devices.	A deep learning model was built using data obtained from different handheld devices that gave better results than the initial creation of a new model.
Noh et al [55], (2021)	63-89 (73)	In total, 3 walking tests were conducted on a 20-meter straight walkway, involving walking at slower, faster, or the participant's preferred speed. Participants were categorized into low fall-risk and high fall-risk groups based on their fall history and fear of falling.	The ML XGB algorithm was applied to perform the predictive analysis. The predictive analysis sought to predict factors affecting the risk of falls. The parameters were spatiotemporal parameters extracted from the gait tests.	In total, 34 characteristics affecting the risk of falls were extracted and classified into high fall-risk and low fall-risk groups. The study demonstrated >70% accuracy. Stride length, speed, and posture accurately classified the risk of falling.
Qiu et al [53], (2018)	nd	Participants underwent 7 tests: Sensory Integration Test, Limits of Stability Test, Sit-to-Stand test (5 times), TUG test, motor function test, Choice Reaction test, and Fear of Falling test.	A total of 38 algorithms were developed to extract meaningful measurements from the data extracted from the sensors. In total, 38 meaningful measures were obtained and incorporated into a supervised ML model. A prediction of “fallers” and “nonfallers” was made based on this data. The accuracy of the prediction was assessed through a 10-fold cross-validation.	The data obtained distinguished between people who fall and those who do not, with overall accuracy of 89.4% (92% sensitivity and 84.9% specificity). The model created from the inertial sensors in the test battery and the subsequent analysis of the data through AI are promising.
Roshdibenam et al [71], (2021)	65-95 (74.4)	A physiotherapist conducted various assessments, including the TUG test, 30-Second Stand Test, and 4-Stage Balance tests, and classified participants into “high” and “low” fall risk categories.	The CNN algorithm was used for the data for each individual sensor location. Algorithms were applied to evaluate the data obtained by the portable sensors.	Deep learning had high similarities to those obtained by the physiotherapist. Deep learning techniques combined with nonintrusive wearable sensors could provide a new tool for measuring risk factors and predicting fall risk.
Tang et al [69], (2022)	nd (66)	Participants were divided into 2 groups: “Normal walking” and “Abnormal gait.” The “abnormal” group had a higher average age and a greater proportion of men compared to the “normal” group. The TUG test videos of all participants were observed.	ML with the AlphaPose algorithm and the naïve Bayes classifier to identify normal or abnormal gait.	The naïve Bayes classifier obtained a complete data accuracy of 90.1% and a LOOCV^r^ accuracy of 89.1% for the detection of abnormal gait performance.
Tunca et al [62], (2020)	nd (76.8)	Participants were divided into 2 groups: “high fall risk” and “low fall risk.” A total of 2 intervention groups were created: the validation set (30 low risk+30 high risk) and the test set (9 low risk+7 high risk). Participants were instructed to walk 8 m in a straight line at least 3 times.	The applicability of deep learning LSTM^s^ networks were compared with traditional ML with respect to fall risk.	The LSTM achieved classification accuracy of 89% compared to accuracy of 82.8% (the highest accuracy achieved by the traditional ML models used).
Ye et al [60], (2020)	65-nd (nd)	A record of falls was compiled from April 2017 to March 2018. A total of 4361 falls were recorded.	The XGB algorithm was used to create the crash prediction model.	The fall prediction model achieved improved discriminatory ability. It may be able to identify risk factors and facilitate prevention interventions.

^a^AI: artificial intelligence.

^b^ML: machine learning.

^c^nd: none described.

^d^ADL: activities of daily living.

^e^TUG: Timed Up and Go.

^f^BBS: Berg Balance Scale.

^g^ASM: associative skill memories.

^h^CNN: convolutional neural network.

^i^FP: fall prevention.

^j^IMU: inertial measurement unit.

^k^AUROC: area under the receiver operating characteristic curve.

^l^AUPR: area under the precision-recall curve.

^m^MFS: Morse Fall Scale.

^n^XGB: extreme gradient boosting.

^o^MCT: motor control test.

^p^GBM: gradient boosting machine.

^q^RF: random forest.

^r^LOOCV: leave-one-out cross-validation.

^s^LSTM: long short-term memory.

### Applied Assessment Procedures

All articles initially analyzed the characteristics of their samples to subsequently create a predictive model of the risk of falls. However, they did not all follow the same evaluation process.

**Figure 2:**
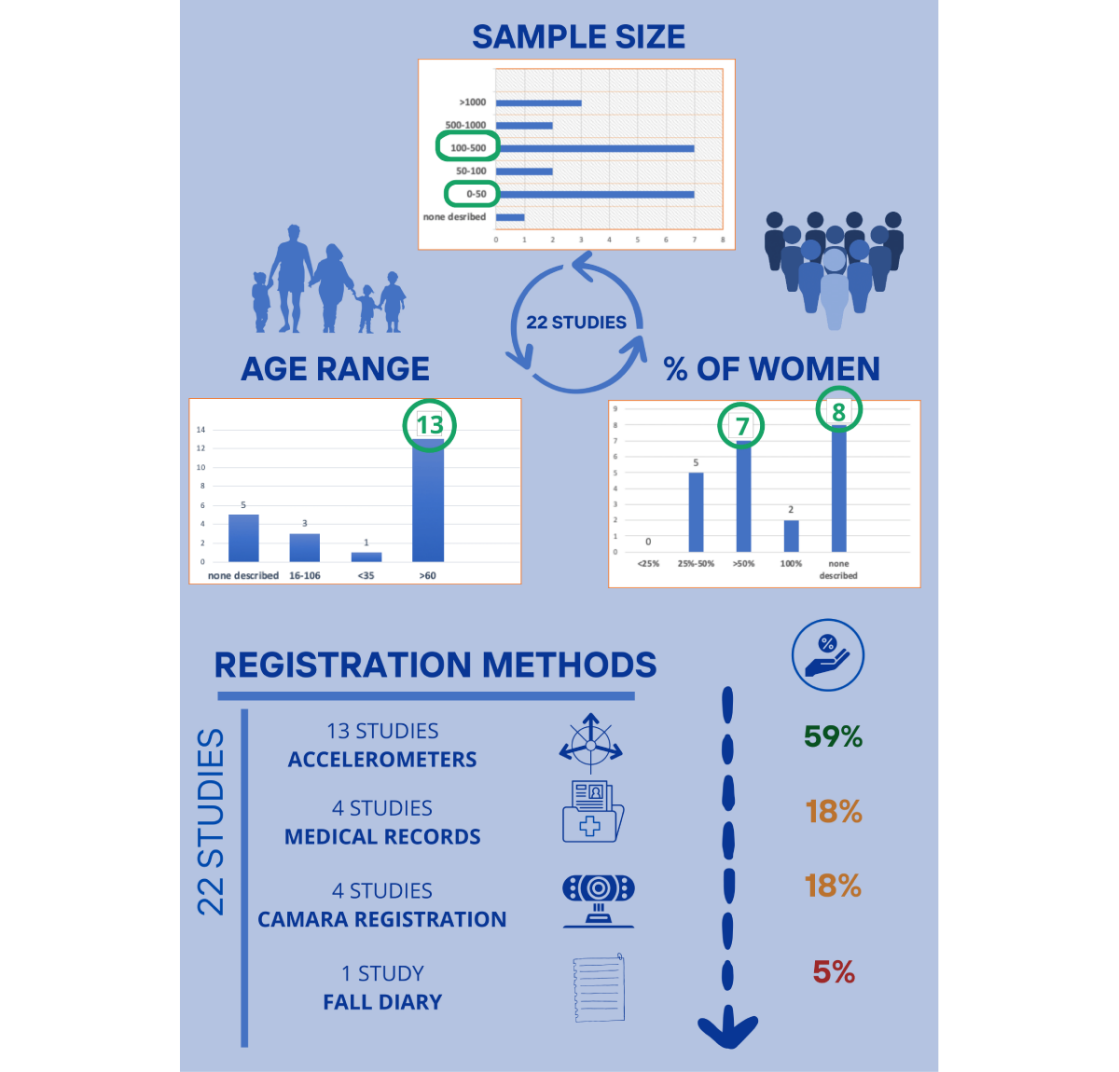
Infographic about the characteristics of the analyzed research.

Regarding the applied assessment procedures, 3 main options stood out: studies with tests or assessments accompanied by sensors or accelerometers [51-57,59,61-63,66,70-72], studies with tests or assessments accompanied by cameras [68,69], or studies based on medical records [58,60,65,67] (Figure 2). Guillan et al [64] performed a physical and functional evaluation of the participants. In their study, they evaluated parameters such as walking speed, stride frequency and length, and the minimum space between the toes. Afterward, they asked them to record the fall events they had during the past 2 years in a personal diary.

In total, 22.7% (5/22) of the studies used the Timed Up and Go test [53,54,69,71,72]. In 18.2% (4/22) of them, the participants performed the test while wearing a sensor to collect data [53,54,71,72]. In 1 (4.5%) study, the test was recorded with a camera for later analysis [69]. Another commonly used method in studies was to ask participants to perform everyday tasks or activities of daily living while a sensor collected data for subsequent analysis. Specifically, 18.2% (4/22) of the studies used this method to gather data [51,56,61,62].

A total of 22.7% (5/22) of the studies asked participants to simulate falls and nonfalls while a sensor collected data [52,61-63,66]. In this way, the data obtained were used to create the predictive model of falls. As for the tests used, Eichler et al [68] asked participants to perform the Berg Balance Scale while a camera recorded their performance.

Finally, other authors created their own battery of tests for data extraction [55,59,64,70]. Gillain et al [64] used gait records to analyze speed, stride length, frequency, symmetry, regularity, and foot separation. Hu et al [59] asked their participants to perform normal walking, the postural reflexive response test, and the motor control test. In the study by Noh et al [55], gait tests were conducted, involving walking 20 m at different speeds. Finally, Greene et al [70] created a 12-question questionnaire and asked their participants to maintain balance while holding a mobile phone in their hand.

### AI Techniques

The selected articles used various techniques within AI. They all had the same objective in applying these techniques, which was to achieve a predictive and classification model for the risk of falls [51-72].

In chronological order, in 2018, Nait Aicha et al [51] compared single-task learning models with multitask learning, obtaining better evaluation results through multitask learning. In the same year, Dubois et al [54] applied AI techniques that analyzed multiple parameters to classify the risk of falls in their sample. Qiu et al [53], also in the same year, used 6 machine learning models (logistic regression, naïve Bayes, decision tree, RF, boosted tree, and support vector machine) in their research.

In contrast, in 2019, Ferrete et al [52] compared the applicability of 2 different deep learning models: the classifier based on associative skill memories and a CNN classifier. In the same year, after confirming the applicability of AI as a predictive method for the risk of falls, various authors investigated through methods such as the RF to identify factors that can predict and quantify the risk of falls [63,65].

Among the selected articles, 5 (22.7%) were published in 2020 [58-62]. The research conducted by Tunca et al [62] compared the applicability of deep learning LSTM networks with traditional machine learning applied to the risk of falls. Hu et al [59] first used cross-validation, where algorithms were trained randomly, and then used the gradient boosting machine algorithm to classify participants as high or low risk. Ye et al [60] and Hsu et al [58] both used the extreme gradient boosting (XGBoost) algorithm based on machine learning to create their predictive model. In the same year, Althobaiti et al [61] trained machine learning models for their research.

In 2021, Lockhart et al [57] started using 3 deep learning techniques simultaneously with the same goal as before: to create a predictive model for the risk of falls. Specifically, they used the RF, RF with feature engineering, and RF with feature engineering and linear and nonlinear variables. Noh et al [55], again in the same year, used the XGBoost algorithm, while Roshdibenam et al [71], on the other hand, used the CNN algorithm for each location of the wearable sensors used in their research. Various machine learning techniques were used for classifying the risk of falls and for balance loss events in the research by Hauth et al [56]. Dubois et al [72] used the following algorithms: decision tree, adaptive boosting, neural net, naïve Bayes, k-nearest neighbors, linear support vector machine, radial basis function support vector machine, RF, and quadratic discriminant analysis. Hauth et al [56], on the other hand, used regularized logistic regression and bidirectional LSTM networks. In the research conducted by Greene et al [70], AI was used, but the specific procedure that they followed is not described.

Tang et al [69] published their research with innovation up to that point. In their study, they used a smart gait analyzer with the help of deep learning techniques to assess the diagnostic accuracy of fall risk through vision. Months later, in August 2022, Ladios-Martin et al [67] published their research, in which they compared 2 deep learning models to achieve the best results in terms of specificity and sensitivity in detecting fall risk. The first model used the Bayesian Point Machine algorithm with a fall prevention variable, and the second one did not use the variable. They obtained better results when using that variable, a mitigating factor defined as a set of care interventions carried out by professionals to prevent the patient from experiencing a fall during hospitalization. Particularly controversial, as its exclusion could obscure the model’s performance. Eichler et al [68], on the other hand, used machine learning–based classifier training and later tested the performance of RFs in score predictions.

Finally, in January 2023, Maray et al [66] published their research, linking the previously mentioned terms (AI and fall risk) with 3 wearable devices that are commonly used today. They collected data through these devices and applied transfer learning to generalize the model across heterogeneous devices.

### Findings

The results of the 22 articles provided promising data, and all of them agreed on the feasibility of applying various AI techniques as a method for predicting and classifying the risk of falls. Specifically, the accuracy values obtained in the studies exceed 70%. Noh et al [55] achieved the “lowest” accuracy among the studies conducted, with a 70% accuracy rate. Ribeiro et al [52] obtained an accuracy of 92.7% when using CNN to differentiate between normal gait and fall events. Hsu et al [58] further demonstrated that the XGBoost model is more sensitive than the Morse Fall Scale. Similarly, in their comparative study, Nait Aicha et al [51] also showed that a predictive model created from accelerometer data with AI is comparable to conventional models for assessing the risk of falls. More specifically, Dubois et al [54] concluded that using 1 gait-related parameter (excluding velocity) in combination with another parameter related to seated position allowed for the correct classification of individuals according to their risk of falls.

## Discussion

### Principal Findings

The aim of this research was to analyze the scientific evidence regarding the applications of AI in the analysis of data related to postural control and the risk of falls. On the basis of the analysis of results, it can be asserted that the following risk factors were identified in the analyzed studies: age [65], daily habits [65], clinical diagnoses [65], environmental and hygiene factors [65], sex [64], stride length [55,72], gait speed [55], and posture [55]. This aligns with other research that also identifies sex [73,74], age [73], and gait speed [75].

On the other hand, the “fear of falling” has been identified in various studies as a risk factor and a predictor of falls [73,76], but it was not identified in any of the studies included in this review.

As for the characteristics of the analyzed samples, only 9.1% (2/22) of the articles used a sample composed exclusively of women [53,59], and no article used a sample composed exclusively of men. This fact is incongruent with reality, as women have a longer life expectancy than men, and therefore, the number of women aged >65 years is greater than the number of men of the same age [77]. Furthermore, women experience more falls than men [78]. The connection between menopause and its consequences, including osteopenia, suggests a higher risk of falls among older women than among men of the same age [79,80].

Within the realm of analysis tools, the most frequently used devices to analyze participants were accelerometers [51-57,59,61-63,66,70-72]. However, only 36.4% (8/22) of the studies provided all the information regarding the characteristics of these devices [51,53,59,61,63,66,70,72]. On the other hand, 18.2% (4/22) of the studies used the term “inertial measurement unit” as the sole description of the devices used [55-57,71].

The fact that most of the analyzed procedures involved the use of inertial sensors reflects the current widespread use of these devices for postural control analysis. These sensors, in general (and triaxial accelerometers in particular), have demonstrated great diagnostic capacity for balance [81]. In addition, they exhibit good sensitivity and reliability, combined with their portability and low economic cost [82]. Another advantage of triaxial accelerometers is their versatility in both adult and pediatric populations [83-86], although the studies included in this review did not address the pediatric population.

The remaining studies extracted data from cameras [68,69], medical records [58,60,65,67], and other functional and clinical tests [59,64,70]. Regarding the AI techniques used, out of the 18.2% (4/22) of articles that used deep learning techniques [52,57,62,71], only 4.5% (1/22) did not provide a description of the sample characteristics [52]. In this case, the authors focused on the AI landscape, while the rest of the articles strike a balance between AI and health sciences.

Regarding the validity of the generated models, only 40.9% (9/22) of the articles assessed this characteristic [52,53,55,61-64,68,69]. The authors of these 9 (N=22, 40.9%) articles evaluated the validity of the models through accuracy. All the results obtained reflected accuracies exceeding 70%, with Ribeiro et al [52] achieving a notable accuracy of 92.7% and 100%. Specifically, they obtained a 92.7% accuracy through the CNN model for distinguishing normal gait, the prefall condition, and the falling situation, considering the step before the fall, and 100% when not considering it [52].

The positive results of sensitivity and specificity can only be compared between the studies of Qiu et al [53] and Gillain et al [64], as they were the only ones to take them into account, and in both investigations, they were very high. Similarly, in the case of the *F*_1_-score, only Althobaiti et al [61] examined this validity measure. This measure is the result of combining precision and recall into a single figure, and the outcome obtained by these researchers was promising.

Despite these differences, the 22 studies obtained promising results in the health care field [51-72]. Specifically, their outcomes highlight the potential of AI integration into clinical settings. However, further research is necessary to explore how health care professionals can effectively use these predictive models. Consequently, future research should focus on studying the application and integration of the already-developed models. In this context, fall prevention plans could be implemented for the target populations identified by the predictive models. This approach would allow for a retrospective analysis to determine if the combination of predictive models with prevention programs effectively reduces the prevalence of falls in the population.

### Limitations

Regarding limitations, the articles showed significant variation in the sample sizes selected. Moreover, even in the study with the largest sample size (with 265,225 participants [60]), the amount of data analyzed was relatively small. In addition, several of the databases used were not generated specifically for the published research but rather derived from existing medical records [58,60,65,67]. This could explain the significant variability in the variables analyzed across different studies.

### Strengths

Despite the limitations, this research has strengths, such as being the first systematic review on the use of AI as a tool to analyze postural control and the risk of falls. Furthermore, a total of 6 databases were used for the literature search, and a comprehensive article selection process was carried out by 3 researchers. Finally, only cross-sectional observational studies were selected, and they shared the same objective.

### Conclusions

The use of AI in the analysis of data related to postural control and the risk of falls proves to be a valuable tool for creating predictive models of fall risk. It has been identified that most AI studies analyze accelerometer data from sensors, with triaxial accelerometers being the most frequently used.

For future research, it would be beneficial to provide more detailed descriptions of the measurement procedures and the AI techniques used. In addition, exploring larger databases could lead to the development of more robust models.

## Appendix

Multimedia Appendix 1 Quality scores of reviewed studies (Critical Review Form for Quantitative Studies tool results).

Multimedia Appendix 2 PRISMA (Preferred Reporting Items for Systematic Reviews and Meta-Analyses) checklist.

## Abbreviations

AI: artificial intelligence
CNN: convolutional neural network
LSTM: long short-term memory
MeSH: Medical Subject Headings
RF: random forest
ROBINS-E: Risk of Bias in Nonrandomized Studies of Exposures
