# Supplementary material for: The Applications of Artificial Intelligence for Assessing Fall Risk: Systematic Review
Source: J Med Internet Res. 2024 Apr 29;26:e54934. doi: 10.2196/54934 (PMC11091813; doi:10.2196/54934)
Supplement: Multimedia Appendix 1 [file jmir_v26i1e54934_app1.docx]

| Table S1. Quality scores of reviewed studies (Critical Review Form for Quantitative Studies tool results) | | | | | | | | | | |  |  |
| --- | --- | --- | --- | --- | --- | --- | --- | --- | --- | --- | --- | --- |
|  | **Aicha et al.^51^ (2018)** | **Althobaiti et al.^61^ (2020)** | **Dubois et al.^54^ (2018)** | **Dubois et al.^72^ (2021)** | **Eichler et al.^69^ (2022)** | **Ferrete et al.^52^ (2019)** | **Gillain et al.^64^ (2019)** | **Greene et al.^70^(2021)** | **Hauth et al.^56^ (202)** | **Hsu et al.^58^ (2020)** | **Hu et al.^59^ (2020)** | **Kim et al.^63^ (2019)** |
| Purpose clearly stated | **✔** | **✔** | **✔** | **✔** | **✔** | **✔** | **✔** | **✔** | **✔** | **✔** | **✔** | **✔** |
| Relevant literature review | **✔** | **✔** | **✔** | **✔** | **✔** | **✔** | **✔** | **✔** | **✔** | **✔** | **✔** | **✔** |
| Study design appropriate to study aims | **✔** | **✔** | **✔** | **✔** | **✔** | **✔** | **✔** | **✔** | **✔** | **✔** | **✔** | **✔** |
| Sample described in detail | **✔** | **✔** | **✔** | **✔** | **X** | **X** | **✔** | **✔** | **X** | **X** | **X** | **X** |
| Sample size justified | **✔** | **✔** | **✔** | **✔** | **✔** | **X** | **✔** | **✔** | **✔** | **✔** | **✔** | **X** |
| Informed consent gained and stated | **X** | **✔** | **✔** | **✔** | **✔** | **X** | **✔** | **✔** | **✔** | **✔** | **✔** | **✔** |
| No biases present | **✔** | **✔** | **✔** | **✔** | **✔** | **✔** | **✔** | **✔** | **✔** | **✔** | **✔** | **✔** |
| Reported using valid outcome measures | **✔** | **✔** | **✔** | **✔** | **✔** | **✔** | **✔** | **✔** | **✔** | **✔** | **✔** | **✔** |
| Reported using reliable outcome measures | **✔** | **✔** | **✔** | **✔** | **✔** | **X** | **✔** | **✔** | **✔** | **✔** | **✔** | **✔** |
| Intervention described in detail | **✔** | **✔** | **✔** | **✔** | **✔** | **X** | **✔** | **✔** | **✔** | **✔** | **✔** | **✔** |
| Statistical reporting of results | **✔** | **✔** | **✔** | **✔** | **✔** | **✔** | **✔** | **✔** | **✔** | **✔** | **✔** | **✔** |
